# Entropy Evolution in Consensus Networks

**DOI:** 10.1038/s41598-017-01615-5

**Published:** 2017-05-08

**Authors:** Shuangshuang Fu, Guodong Shi, Ian R. Petersen, Matthew R. James

**Affiliations:** 1 School of Mathematics and Physics, University of Science and Technology Beijing, Beijing, 100083 China; 2 Research School of Engineering, The Australian National University, Canberra, 0200 Australia

## Abstract

We investigate the evolution of the network entropy for consensus dynamics in classical and quantum networks. We show that in the classical case, the network differential entropy is monotonically non-increasing if the node initial values are continuous random variables. While for quantum consensus dynamics, the network’s von Neumann entropy is in contrast non-decreasing. In light of this inconsistency, we compare several distributed algorithms with random or deterministic coefficients for classical or quantum networks, and show that quantum algorithms with deterministic coefficients are physically related to classical algorithms with random coefficients.

## Introduction

How agreement emerging among a group of agents interacting each other is an intriguing subject in various research disciplines^1–5^. The fundamental idea lies in that, cooperative decisions can lead to consensus in node states throughout a network even each node can only interact with a few neighboring nodes. Inspired by this, consensus control over networks has been systematically studied in the past decade^6, 7^, becoming one of the foundational blocks for engineering solutions to distributed coordination problems in multi-agent systems^8^. Recent work^9, 10^, further developed consensus dynamics for quantum networks, where each node corresponds to a qubit^11^. The concepts regarding the network density matrix as statistical ensembles of pure quantum states, reaching a quantum consensus were systematically developed^9^, and it has been shown that a quantum consensus can be reached with the help of quantum swapping operators for both continuous-time and discrete-time dynamics^9, 12^. In fact, the two categories of dynamics over classical and quantum networks can be put together into a group-theoretic framework^10^, and quantum consensus dynamics can in fact be equivalently mapped into certain parallel classical dynamics over disjoint subsets of the entries of the network density matrix^12^. This line of research on consensus dynamics is related to the work on quantum walks over complex networks^13, 14^, where associated with the network Laplacian there is a Hermitian operator defining the evolution of the network state in a quantum space.

Despite of their algorithmic consistency from a high level between classical and quantum consensus dynamics^10, 12^, it is worth investigating the two categories of physical processes from an information perspective. Classical consensus dynamics is realized by nodes observing their neighbors’ states (or, their relative states) and then taking real-time feedback. On the other hand, such precise state observation among different components in a quantum network is proven to be impossible^11^, and quantum consensus dynamics is realized via nodes interacting not directly, but with the help of local environments which by themselves are quantum subsystems. Tracing out these local environments, the state evolution of the quantum network follows a master equation with the same attraction nature^12^.

To this end, we investigate the evolution of the network entropy for consensus dynamics in classical and quantum networks. We show that in classical consensus dynamics, the network network differential entropy is monotonically non-increasing if the node initial values are continuous random variables with proper density functions. While for quantum consensus dynamics, the network’s von Neumann entropy is in contrast non-decreasing. These observations suggest that the two types of consensus schemes have different physical footings. Then, we compare several gossiping algorithms with random or deterministic coefficients for classical or quantum networks and present novel convergence conditions for gossiping algorithms with random coefficients. The result shows that quantum gossiping algorithms with deterministic coefficients are physically consistent with classical gossiping algorithms with random coefficients.

## Results

Consider a network with *N* nodes. The nodes are indexed in the set V = {1, …, *N*}, and their interconnections are described by a connected undirected graph G = (V, E), where each element in E is an unordered pair of nodes in V. The graph G is an abstraction to the components (given by V) and the structure (given by E) of the information flow among the components.

### Classical Networks

In classical scenarios, each node *i* *∈* V holds a real-valued state at time *t*, denoted *X*_*i*_(*t*), representing opinions of a peer in social networks, or signals measured at a sensor in engineering networks. The network state is represented by the vector $X(t)={({X}_{1}(t)\ldots {X}_{N}(t))}^{{\rm{T}}}$. The Laplacian of the graph G is an *N* × *N* matrix defined by *L*_G_ = *D*_G_ − *A*_G_, where A_G_ is the adjacency matrix of G with [*A*_G_]_*ij*_ = 1 if {*i*, *j*} ∈ E and [*A*_G_]_*ij*_ = 0 otherwise, and *D*_G_ is a diagonal matrix with the $i$ th diagonal entry given by the degree of node *i*, ${d}_{i}={\sum }_{j}{[{A}_{{\rm{G}}}]}_{ij}$. In continuous-time settings, DeGroot’s type of node interactions lead to a time evolution of *X*(*t*) described by the following differential equation (7)

1 $$\frac{d}{dt}X(t)=-\,{L}_{{\rm{G}}}X(t\mathrm{).}$$

The initial time is set to be 0 and each node *i* holds an initial value *X*_*i*_(0). During (1) the nodes’ states are mixed in such a way that at each time *t*, the direction of the movement of *X*_*i*_(*t*) always points to the interior of the convex combination of its neighbors’ current states. In the end an average consensus is reached in the sense that (7)

$$X(\infty )\,:\,=\mathop{\mathrm{lim}}\limits_{t\to \infty }X(t)={\bf{1}}{{\bf{1}}}^{{\rm{T}}}X\mathrm{(0)}/N$$

where **1** is the *N* × 1 vector with all entries being **1**.

The Shannon entropy is a fundamental measure of uncertainty of a random variable^15^. The entropy *H*(*Z*), of a discrete random variable Z with alphabet ${\mathscr{Z}}$ is defined as $H(Z)\,:\,=-{\sum }_{z\in {\mathscr{Z}}}p(z)\mathrm{log}\,p(z\mathrm{).}$ Here log is to the base 2 and *p*(·) is the probability mass function. The differential entropy h(*Z*) of a continuous random variable *Z* with density *f*(*z*) is defined as $h(Z)\,:\,=-\,{\int }_{{\mathscr{S}}}f(z)\mathrm{log}\,f(z)dz$, where ${\mathscr{S}}$ is the support of *Z*. Then the following result holds.

**Theorem 1** *Let the X*_*i*_(0) *be continuous random variables. Then* $h(X(t))\ge h(X(s))$ for all $0\le t\le s$ along the system (1).

When the *X*_*i*_(0) are independent and identically distributed (i.i.d.) discrete random variables, the Shannon entropy of *X*(*t*), $H(X(t))$, is invariant for all $t\ge 0$ along the system (1) since there is a one-to-one mapping between $X(t+\varepsilon )$ and *X*(*t*) for any *t* > 0 and $\varepsilon  > 0$ from the proof of Theorem 1. We also note that the entropy at individual nodes, either $h({X}_{i}(t))$ or $H({X}_{i}(t))$, can certainly admit non-monotone trajectories. Particularly, if the *X*_*i*_(0) are i.i.d. Gaussian with mean *μ* and variance σ^2^, we know

$$h({X}_{i}\mathrm{(0))}=\frac{1}{2}[\mathrm{log}\,\mathrm{(2}\pi e{\sigma }^{2})],\,h({X}_{i}(\infty ))=\frac{1}{2}\,\mathrm{log}\,\mathrm{(2}\pi e{\sigma }^{2}/N)$$

since ${X}_{i}(\infty )$ is also a Gaussian random variable but with mean *μ* and variance ${\sigma }^{2}/N$. On the other hand, if the *X*_*i*_(0) are i.i.d. Bernoulli random variables with mean $p\in \mathrm{(0,}\,\mathrm{1)}$, $N{X}_{i}(\infty )$ obeys binomial distribution and therefore

$$H(X\mathrm{(0))}=N[p\,\mathrm{log}\,{p}^{-1}+\mathrm{(1}-p)\mathrm{log}\,{\mathrm{(1}-p)}^{-1}],\,\,H(X(\infty ))\simeq \frac{1}{2}\,\mathrm{log}\,\mathrm{(2}\pi eNp\mathrm{(1}-p))+O(\frac{1}{N})\mathrm{.}$$

### Quantum Networks

In a simple quantum network, each node $i\in {\rm{V}}$ represents a qubit (quantum bit). The state space associated with any isolated quantum system is a complex vector space with inner product, i.e., a Hilbert space ${\mathcal H}$. The system is completely described by its state vector, which is a unit vector in the system’s state space and often denoted by $|\psi \rangle \in  {\mathcal H}$ (known as the Dirac notion). The state space of a composite quantum system is the tensor product of the state space of each component system. Two quantum systems with state spaces ${ {\mathcal H} }_{A}$ and ${ {\mathcal H} }_{B}$, respectively, form a composite system with state space ${ {\mathcal H} }_{A}\otimes { {\mathcal H} }_{B}$, where $\otimes$ stands for tensor product. If the two quantum systems are isolated respectively with states $|{\psi }_{A}\rangle \in { {\mathcal H} }_{A}$ and $|{\psi }_{B}\rangle \in { {\mathcal H} }_{B}$, the composite system admits a state $|{\psi }_{A}\rangle \otimes |{\psi }_{B}\rangle$. Quantum states can also be described by a positive (i.e., positive semi-definite) Hermitian density operator $\rho$ over the Hilbert space. A quantum state $|\psi \rangle \in  {\mathcal H}$, induces a linear operator, denoted $|\psi \rangle \langle \psi |$, by $|\psi \rangle \langle \psi |(|x\rangle )=(\langle \psi |x\rangle )|\psi \rangle$ where $\langle \psi |x\rangle$ is the inner product equipped by the Hilbert space ${\mathcal H}$ with 〈*ψ*| being the dual vector of |*ψ*〉. In this way the operator $\rho =|\psi \rangle \langle \psi |$ describes the quantum system at state |*ψ*〉. Density operators provide a convenient description of *mixed states* as ensembles of pure states: A quantum system in state |*ψ*_*i*_〉 with probability *p*_*i*_ can be described by $\rho ={\sum }_{i}{p}_{i}|{\psi }_{i}\rangle \langle {\psi }_{i}|$. Any positive and Hermitian operator with trace one defines a proper density operator describing certain quantum state, and vice versa.

The state of each qubit is represented by a density operator over the two-dimensional Hilbert space ${\mathcal H}$, and the network state corresponds to a density operator over ${ {\mathcal H} }^{\otimes N}$, the *N*’th tensor product of ${\mathcal H}$. Let $\rho (t)$ be the network density operator at time $t$. The *swapping operator* between qubits *i* and *j*, denoted as *U*_*ij*_, is defined by ${U}_{ij}({q}_{1}\otimes \ldots \otimes {q}_{i}\otimes \ldots \otimes {q}_{j}\otimes \ldots \otimes {q}_{n})={q}_{1}\otimes \ldots \otimes {q}_{j}\otimes \ldots \otimes {q}_{i}\otimes \ldots \otimes {q}_{n}$ for all ${q}_{i}\in  {\mathcal H} ,i=\mathrm{1,}\ldots ,n$. In other words, the swapping operator ${U}_{ij}$ switches the information held on qubits *i* and *j* without changing the states of other qubits. Continuous-time quantum consensus process can be specified by a master equation^11^

2 $$\frac{d}{dt}\rho (t)=\sum _{\{j,k\}\in {\rm{E}}}({U}_{jk}\rho (t){U}_{jk}-\rho (t\mathrm{)).}$$

For the system (2), there holds^9, 12^,

$$\rho (\infty )\,:\,=\mathop{\mathrm{lim}}\limits_{t\to \infty }\rho (t)=\frac{1}{N!}\sum _{\pi \in {\mathfrak{P}}}{U}_{\pi }\rho \mathrm{(0)}{U}_{\pi }^{\dagger }$$

where ${\mathfrak{P}}$ is the permutation group over V, and *U*_*π*_ represents the quantum permutation operator induced by $\pi \in {\mathfrak{P}}$ satisfying ${U}_{\pi }({q}_{1}\otimes \ldots \otimes {q}_{n})={q}_{\pi \mathrm{(1)}}\otimes \ldots \otimes {q}_{\pi (n)}$ for all ${q}_{i}\in  {\mathcal H}$ with $i=1,\ldots ,\,n$.

As a natural generalization of the Shannon entropy, for a quantum-mechanical system described by a density matrix *ρ*, the von Neumann entropy is defined as ref. 11 $S(\rho )=-\,{\rm{tr}}(\rho \,\mathrm{log}\,\rho ),$ where ${\rm{tr}}(\cdot )$ is the trace operator. The following result holds.

**Theorem 2** For the system (2), *S*(*ρ*(*t*) is a non-decreasing function over $\mathrm{[0,}\infty )$.

**Figure 1:**
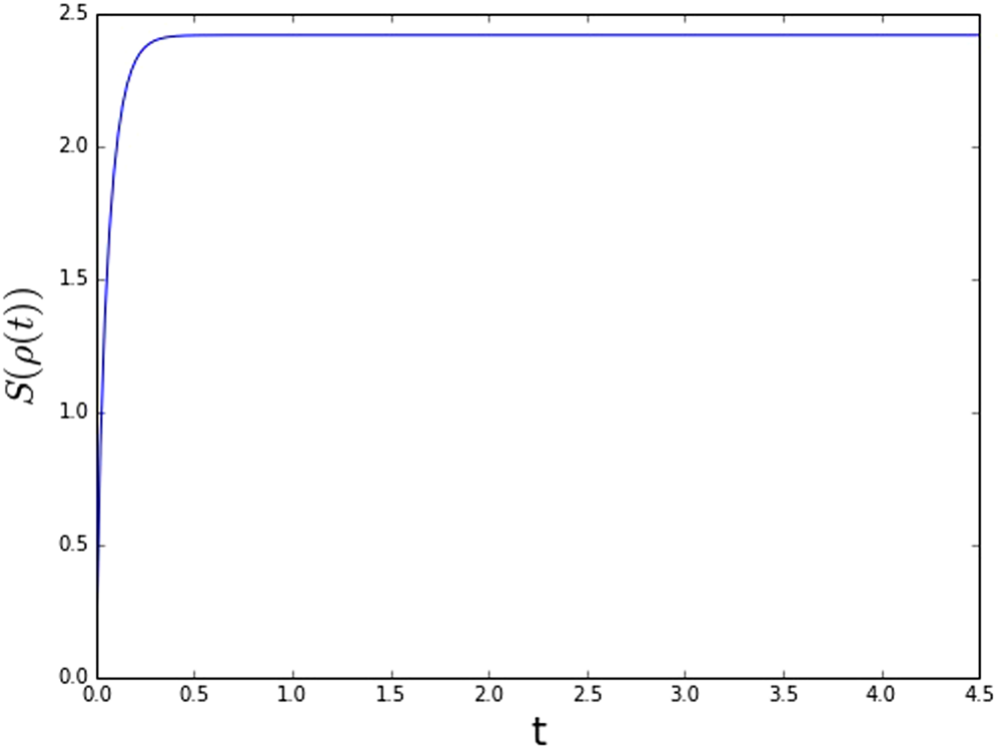
The evolution of network entropy for classical network dynamics.

**Figure 2:**
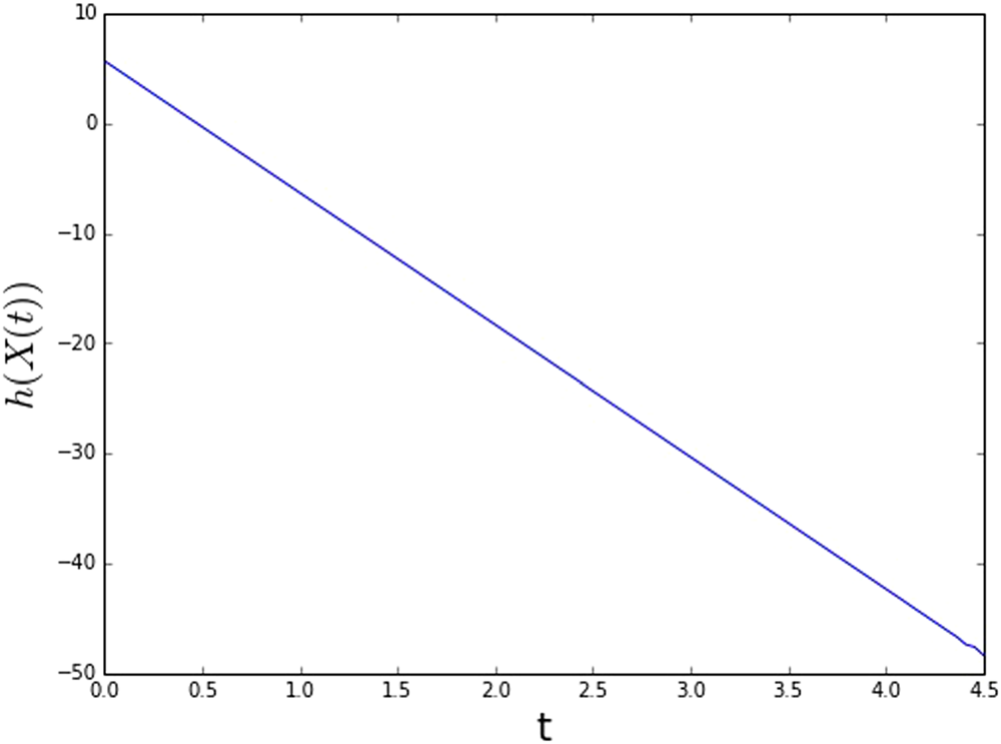
The evolution of network entropy for quantum network dynamics.

**Example**. Let G be a complete graph with 4 nodes. For the classical case, we take the *X*_*i*_(0) as an i.i.d. standard Gaussian random variable. For the quantum case, we take the initial density matrix as $\rho \mathrm{(0)}=|01+-\rangle \langle 01+-|\mathrm{.}$ The evolution of the differential entropy and the von Neumann entropy with the classical and quantum consensus dynamics is plotted, respectively, in Figs 1 and 2.

### Algorithmic and Physical Equivalence

The above results reveal that, the network entropy in general decreases with classical consensus dynamics, but increases with quantum consensus dynamics. This appears to be surprising noticing their consistencies pointed out in refs 9 and 12. However, although the systems (1) and (2) can be formally united from an algorithmic point of view (cf. ref. 12), *X*(*t*) represents a random variable in the classical world, while *ρ*(*t*) is a probability mass function by its definition. We now provide a *physical* perspective to explain the observations in Theorem 1 and Theorem 2 by investigating a serial of classical or quantum gossiping algorithms with random or deterministic coefficients.

A random gossiping process is defined as follows. Consider *N* nodes in the set V with an underlying interaction graph G which is undirected and connected. Time is sequenced by $k=0,1,\ldots$. At time *k*, a node *i* is first drawn with probability 1/*N*, and then node *i* selects another node *j* who shares a link with node *i* in the graph G with probability $\mathrm{1/}{\rm{\deg }}(i)$. Here ${\rm{\deg }}(i)$ is the degree of node *i* in the graph V. In this way, a random pair $\{i,j\}$ is selected. Additionally, let ${b}_{k},k=0,1,\ldots$ be a sequence of i.i.d. Bernoulli random variables with mean 1/2, which are also independent of any other possible randomness.

- In the classical case, each node *i* holds a real-valued state ${X}_{i}(k)\in R$ at time *k*. Their initial states, ${X}_{1}\mathrm{(0),}\ldots ,{X}_{N}\mathrm{(0)}$, are assumed to be *N* (not necessarily independent, continuous or discrete) random variables over a common underlying probability space. The marginal probability (mass or density) distribution of node ${X}_{i}(k)$ is denoted as ${p}_{k}^{i}(\,\cdot \,)$. When the pair $\{i,j\}$ is selected at time *k*, only the two selected nodes update their values and we consider the following algorithms.
  [A1] (*Classical Gossiping with Deterministic Coefficients* ^16^) Node *i* and *j* update their values as
  3 $${X}_{i}(k+\mathrm{1)=}{X}_{j}(k+\mathrm{1)}\,=\,\frac{1}{2}{X}_{i}(k)+\frac{1}{2}{X}_{j}(k\mathrm{).}$$
  [A2] (*Classical Gossiping with Random Swapping* ^17^) Node *i* and *j* update their values as
  4 $${X}_{i}(k+\mathrm{1)}\,=\,{b}_{k}{X}_{i}(k)+\mathrm{(1}-{b}_{k}){X}_{j}(k);\,{X}_{j}(k+\mathrm{1)}\,=\,\mathrm{(1}-{b}_{k}){X}_{i}(k)+{b}_{k}{X}_{j}(k\mathrm{).}$$
- In the quantum case, each node $i$ represents a qubit and $\rho (k)$ is the network density matrix at time $k$. When the qubit pair $\{i,j\}$ is selected at time $k$, we correspondingly consider the following algorithms.

[AQ1] (*Quantum Gossiping with Deterministic Coefficients* ^9^) The quantum network updates its density matrix as

5 $$\rho (k+\mathrm{1)}=\frac{1}{2}\rho (k)+\frac{1}{2}{U}_{ij}\rho (k){U}_{ij}^{\dagger }\mathrm{.}$$

[AQ2] (*Quantum Gossiping with Random Swapping* ^10^) Node *i* and *j* update their values as

6 $$\rho (k+\mathrm{1)}={b}_{k}\rho (k)+\mathrm{(1}-{b}_{k}){U}_{ij}\rho (k){U}_{ij}^{\dagger }\mathrm{.}$$

We state a few immediate facts for the algorithms [A1], [A2], [AQ1], and [AQ2].

(i) The evolution of ${\bf{E}}\{X(k)\}$ is exactly the same along with the algorithms [A1] and [A2]. Similar conclusion holds also for the algorithms [AQ1] and [AQ2].
(ii) Algorithms [A1] and [AQ1] are *algorithmically equivalent*, in the sense that [AQ1] can be divided into a set of parallel algorithms in the form of [A1] over disjoint entries of $\rho (t)$ (see ref. 12 for a thorough treatment via vectorizing $\rho (t)$). Similarly, the algorithms [A2] and [AQ2] are algorithmically equivalent.
(iii) Algorithms [A2] and [AQ1] are *physically equivalent*, in the sense that for a sequence of underlying random variables $X(k)$ evolving along [A2], their joint probability mass/density function, denoted ${f}_{k}({x}_{1},\ldots ,{x}_{N})$ (which is exactly the physical interpretation of the density matrix $\rho (k)$) will evolve in the form of [AQ1] (cf., ref. 10):

7 $${f}_{k+1}({x}_{1},\ldots ,{x}_{N})=\frac{1}{2}{f}_{k}({x}_{1},\ldots ,{x}_{i},\ldots ,{x}_{j},\ldots ,{x}_{N})+\frac{1}{2}{f}_{k}({x}_{1},\ldots ,{x}_{j},\ldots ,{x}_{i},\ldots ,{x}_{N})$$

if the pair $\{i,j\}$ is selected at time $k$.

Recall that a Markov chain is *irreducible* if there exists a positive probability that the chain transits from one state to another in some finite steps between any pair of states; *aperiodic* if the greatest common divisor of the minimal steps admitting a positive probability from any state to itself is exactly one; *ergodic* if it is both aperiodic and irreducible^18^. We present the following result establishing the limiting behaviors of the algorithm [A2], which is consistent with the observations of the entropy evolution in Theorems 1 and 2 as well as the point (iii) above.

**Theorem 3** *For the algorithm* [*A2*], *the following statements hold*. (*i*) ${\{X(k)\}}_{k\mathrm{=0}}^{\infty }$ *forms an ergodic Markov chain given X*(0); (ii) ${\mathrm{lim}}_{k\to \infty }{p}_{k}^{i}(\,\cdot \,)={\sum }_{i\mathrm{=1}}^{N}{p}_{0}^{i}(\,\cdot \,)/N$, where the convergence is exponentially fast under the distance induced by ${\ell }^{1}$ (for *X*(0) given by discrete random variables) or ${ {\mathcal L} }^{1}$ (for continuous *X*(0) norms; (iii) the network entropy (continuous or discrete) is non-decreasing.

Note that Theorem 1 indeed shows that the network entropy along the algorithm [A1] is non-increasing in the limit for certain classes of initial distributions. It is reasonable to believe that such non-increasing entropy holds in general for the algorithm [A1], although establishing a rigorous proof would be challenging. In the algorithms [A1] and [A2], the randomness of *X*(*k*) comes from three resources: initial random draws *X*(0), random pair selections, and external randomness in the updates (the Bernoulli random variables *b*_*k*_). It is clear that we can assume a deterministic pair sequence in their updates, all the above conclusions in Theorems 1 and 3 continue to hold as long as this deterministic sequence visits each pair in the graph sufficiently often. It now becomes clear that it is the external randomness in [A2] driven by {*b*_*k*_} that causes the increasing of network entropy.

One can also consider the case in a gossiping process when two selected node *i* and *j* update their values by (Classical Gossiping with Random Coefficients)

8 $$[A1^{\prime} ]\,{X}_{i}(k+\mathrm{1)}={X}_{j}(k+\mathrm{1)}={b}_{k}{X}_{i}(k)+\mathrm{(1}-{b}_{k}){X}_{j}(k\mathrm{).}$$

From the second Borel-Cantelli Lemma (e.g., Theorem 2.3.6. in ref. 18), that almost surely, *X*_*i*_(*k*) reaches a common value for all $i\in {\rm{V}}$ in finite time along the algorithm [A1′]. Interestingly, it is easy to see that the evolution of the ${p}_{k}^{i}(\,\cdot \,)$ is the same along the algorithms [A1′] and [A2].

The scheme of the algorithms [A2] was briefly discussed in Section 6.2 of ref. 10, which is also a form of gossiping algorithms with unreliable but perfectly dependent link communications studied in ref. 17 with mixing coefficient one. Here Theorem 3 advances the previous understandings by showing that the algorithm [A2] defines an ergodic Markov chain for any given initial condition as well as presenting the detailed convergence properties of the marginal distribution functions for both discrete and continuous *X*(0). Moreover, we assume that the mean of the *b*_*k*_ is 1/2 just for the ease of presentation. It is clear from the proof that Theorem 3 holds for arbitrary ${\bf{E}}\{{b}_{k}\}\in \mathrm{(0,1)}$. The ergodicity plays an essential role in the convergence of the marginal distributions: the case with ${\bf{E}}\{{b}_{k}\}=0$ fails because *X*(*k*) is no longer aperiodic; the case with ${\bf{E}}\{{b}_{k}\}=1$ fails because *X*(*k*) is no longer irreducible.

## Methods

This section provides the proofs of Theorems 1, 2 and 3.

**Proof of Theorem 1**

The solution *X*(*t*) of the system (1) is $X(t)={e}^{-t{L}_{{\rm{G}}}}X\mathrm{(0)}$. We take $\varepsilon  > 0$ and compare $h(X(t+\varepsilon ))$ with $h(X(t))$. From

$$X(t+\varepsilon )={e}^{-\varepsilon {L}_{{\rm{G}}}}X(t),$$

we know (Themrem 8.6.4^15^)

$$h(X(t+\varepsilon ))=h(X(t))+\,\mathrm{log}\,|det({e}^{-\varepsilon {L}_{{\rm{G}}}})|\mathrm{.}$$

Since *L*_G_ is the Laplacian of a connected undirected graph G, *L*_G_ has a unique zero eigenvalue, and all non-zero eigenvalues of *L*_G_ are positive^8^. Consequently, all eigenvalues of ${e}^{-\varepsilon {L}_{{\rm{G}}}}$ are positive and no larger than one, which yields that $|det({e}^{-\varepsilon {L}_{{\rm{G}}}})|\le 1.$ This proves $h(X(t+\varepsilon ))\le h(X(t))$. Since $\varepsilon$ is chosen arbitrarily, Theorem 1 holds.  □

**Proof of Theorem 2**

Fix $s\ge 0$. Define a set ${{\rm{\Sigma }}}_{s}={\rm{co}}({U}_{\pi }\rho (s){U}_{\pi }^{\dagger }:\pi \in {\mathfrak{P}}),$ where co(·)stands for the convex hull. It is straightforward to see that ${U}_{jk}\rho {U}_{jk}^{\dagger }\in {{\rm{\Sigma }}}_{s}$ if $\rho \in {{\rm{\Sigma }}}_{s}$. As a result, ${{\rm{\Sigma }}}_{s}$ is an invariant set of the system (2) in the sense that $\rho (t)\in {{\rm{\Sigma }}}_{s}$ for all $t\ge 0$ as long as $\rho \mathrm{(0)}\in {{\rm{\Sigma }}}_{s}$. Therefore, for the system (2) and for any $\varepsilon  > 0$, there exist ${m}_{\pi }(\varepsilon )\ge \mathrm{0,}\,\pi \in {\mathfrak{P}}$ with ${\sum }_{\pi \in {\mathfrak{P}}}{m}_{\pi }(\varepsilon )=1$ such that

9 $$\rho (s+\varepsilon )=\sum _{\pi \in {\mathfrak{P}}}{m}_{\pi }(\varepsilon ){U}_{\pi }\rho (s){U}_{\pi }^{\dagger }\mathrm{.}$$

Recalling that the von Neumann entropy *S*(*ρ*) is a concave function of *ρ*, and that $S(\rho )=S(U\rho {U}^{\dagger })$ for any unitary operator *U*, we conclude that

10 $$S(\rho (s+\varepsilon ))=S(\sum _{\pi \in {\mathfrak{P}}}{m}_{\pi }(\varepsilon ){U}_{\pi }\rho (s){U}_{\pi }^{\dagger })\ge \sum _{\pi \in {\mathfrak{P}}}{m}_{\pi }(\varepsilon )S({U}_{\pi }\rho (s){U}_{\pi }^{\dagger })=S(\rho (s))$$

for any $\varepsilon  > 0$ and $s\ge 0$ in light of the fact that *U*_*π*_ is unitary for all $\pi \in {\mathfrak{P}}$. This proves that $S(\rho (t))$ is a non-decreasing function and Theorem 2 holds.□

**Proof of Theorem 3**

(i) First of all it is clear that ${\{X(k)\}}_{k\mathrm{=0}}^{\infty }$ is Markovian from its definition. Recall that ${\mathfrak{P}}$ is the *N*’th permutation group. We denote the permutation matrix associated with $\pi \in {\mathfrak{P}}$ as ${M}_{\pi }$. In particular, the permutation matrix associated with the swapping between *i* and *j* is denoted as ${M}_{{\pi }_{ij}}$. The state transition of ${\{X(k)\}}_{k\mathrm{=0}}^{\infty }$ along the algorithm A2 can be written as ${\bf{P}}({M}_{{\pi }_{ij}}X(k)|X(k))=\mathrm{(1}/\deg (i)+\mathrm{1/}{\rm{\deg }}(j))/N$ for $\{i,j\}\in {\rm{E}}$. Since the graph G is connected, the swapping permutations defined along the edges of G form a generating set of the permutation group ${\mathfrak{P}}$. Consequently, given *X*(0), the set $\{{M}_{\pi }X\mathrm{(0),}\,\pi \in {\mathfrak{P}}\}$ is the state space of $X(k)$, which contains at most *N*! elements. Finally it is straightforward to verify that for any given *X*(0), *X*(*k*) is irreducible (due to the fact that any two states in the above state space can reach each other by a finite number of swapping permutations along the edges of G) and aperiodic (due to the fact that there is a positive probability that $X(k)=X(k+\mathrm{1)}$ when ${b}_{k}=1$), and therefore forms an ergodic Markov chain.
(ii) The statement is in fact a direct consequence from the ergodicity of *X*(*k*). We however need to be a bit more careful since we assume that *X*(0) takes value from an arbitrary (not necessarily discrete) probability space and the *X*(0) are not necessarily independent. We denote the state transition matrix for *X*(*k*) as $P\in {R}^{N\times N}$. We calculate ${p}_{k}^{i}(\,\cdot \,)$ from basic probability equality ${\bf{P}}(A)={\sum }_{s\mathrm{=1}}^{m}{\bf{P}}(A|{C}_{i})$ under ${\sum }_{i\mathrm{=1}}^{N}{\bf{P}}({C}_{i})=1$ and ${\bf{P}}({C}_{i}\cap {C}_{j})=0$, and then immediately obtain ${p}_{k}^{i}(\,\cdot \,)={\sum }_{s\mathrm{=1}}^{N}{e}_{s}^{T}{P}^{k}{e}_{i}{p}_{0}^{s}(\,\cdot \,)$, where *e*_*i*_ is the unit vector with the *i*’th entry being one. It is clear that the above calculation does not rely on *X*(0) being discrete or continuous, and ${p}_{k}^{i}(\,\cdot \,)$ represents probability mass or density function wherever appropriate. From the definition of the algorithm A2, *P* is a symmetric matrix and the ergodicity of *X*(*k*) leads to ${\mathrm{lim}}_{k\to \infty }{P}^{k}={\bf{1}}{{\bf{1}}}^{T}/N$ at an exponential rate.
(iii) This observation follows directly from (7) and the concavity of (continuous or discrete) entropy.

The desired conclusion thus follows.

## Conclusions

We have investigated the evolution of the network entropy for consensus dynamics in classical or quantum networks. In the classical case, the network differential entropy is monotonically non-increasing with continuous initial random values. For quantum consensus dynamics, the network’s von Neumann entropy is on the contrary non-decreasing. This observation can be easily generalized to balanced directed graphs^19^. In light of this inconsistency, we also compared several gossiping algorithms with random or deterministic coefficients for classical or quantum networks, and showed that quantum gossiping algorithms with deterministic coefficients are physically consistent with classical gossiping algorithms with random coefficients.
